# Juvenile Idiopathic Arthritis and COVID-19 Pandemic: Good Compliance With Treatment, Reluctance to Return to School

**DOI:** 10.3389/fmed.2021.743815

**Published:** 2021-11-12

**Authors:** Baptiste Quéré, Irene Lemelle, Anne Lohse, Pascal Pillet, Julie Molimard, Olivier Richer, Christelle Sordet, Véronique Despert, Linda Rossi-Semerano, Charlotte Borocco, Isabelle Kone-Paut, Elisabeth Gervais, Dewi Guellec, Valérie Devauchelle-Pensec

**Affiliations:** ^1^Department of Rheumatology, Cavale Blanche Hospital, Brest University, Brest, France; ^2^Paediatric Onco-Haematology, Brabois Hospital, University Hospital of Nancy, Vandoeuvre-Lès-Nancy, France; ^3^Department of Rheumatology, Nord Franche-Comté Hospital, Belfort, France; ^4^Paediatrics, Rheumatology and Paediatric Internal Medicine, Children's Hospital, Bordeaux, France; ^5^Department of Rheumatology, University Hospital of Strasbourg, Strasbourg, France; ^6^Department of Paediatric, Children's Hospital, Rennes, France; ^7^Department of Paediatric Rheumatology, National Reference Centre for Auto-inflammatory Diseases and Amyloidosis of Inflammatory Origin (CEREMAIA), Assistance Publique-Hôpitaux de Paris (AP-HP), University of Paris Sud Sacaly, Le Kremlin-Bicêtre, France; ^8^Department of Rheumatology, University Hospital of Poitiers, Poitiers, France; ^9^Department of Rheumatology, Cavale Blanche Hospital, Brest University, INSERM UMR 1227, Brest, France

**Keywords:** juvenile idiopathic arthritis, COVID-19, compliance, treatment, DMARDs, school

## Abstract

**Objective:** The SARS-CoV-2 pandemic has induced an exceptional sanitary crisis, potentially having an impact on treatment continuation, for juvenile idiopathic arthritis (JIA) patients receiving immunosuppressive therapies. After national lockdowns, many patients were also concerned about their safety at school. We evaluated the impact of the pandemic on the optimal continuation of treatment and on the return to school in JIA patients.

**Methods:** JIA patients under 18 years of age, usually treated with disease-modifying anti-rheumatic drugs (DMARDs) were prospectively included during their outpatient visit and completed a standardized questionnaire. The primary outcome was DMARD treatment modification in relation to the context of the pandemic but we also evaluated the pandemic's impact on the schooling.

**Results:** One hundred and seventy three patients from 8 different expert centers were included between May and August 2020. Their mean age was 11.6 years (± 4.1 years), and most of them 31.2% (54/173) had a rheumatoid factor-negative polyarticular JIA. Fifty percent (86/172) were treated with methotrexate, and 72.5% (124/171) were treated with bDMARDs. DMARD treatment modification in relation to the pandemic was observed in 4.0% (7/173) of participants. 49.1% (81/165) of the patients did not return to school due to a personal/parental decision in 69.9% (55/81) of cases. Two patients were diagnosed positive for SARS-CoV-2 infection.

**Conclusion:** This study suggests that JIA patients treated with DMARDs continued their treatment during the pandemic and were rarely affected by symptomatic COVID-19. In contrast, parents' reluctance was a major obstacle for returning to school. Therefore, more solidified school reopening strategies should be developed.

## Introduction

Coronavirus disease 2019 (COVID-19) is a newly recognized illness caused by the SARS-CoV-2 virus (severe acute respiratory syndrome coronavirus 2), which was officially considered a pandemic in March 2020 (1–4). Most SARS-CoV-2 infections lead to moderate influenza-like symptoms, loss of taste or isolated mild fever but can also lead to severe hypoxemic syndrome and death. Data concerning children with COVID-19 seem reassuring, and very few of them develop severe forms of the disease (2, 5–8). In France, the first patients were identified on the 24th of January. Two areas of intense viral circulation, also called hot spots, appeared in February 2020: the first area in the eastern part of France and the second area in Paris. The first lockdown period occurred from 17 March to 11 May, which included the closure of schools and universities. After 11 May, a progressive return to school was then proposed, with many difficulties and fears, first in primary school and then in secondary school. Compulsory return to school was scheduled for June 22nd. The reopening plan to prevent contamination was based in France on physical distancing, face coverings after the age of 6 years, hygiene, staggering of schedules, and smaller fixed groups of students. This happened in a context of general anxiety.

Juvenile idiopathic arthritis (JIA) is a rare disease with different subtypes (9, 10). Patients are treated, in most cases, with immunosuppressant agents but also with nonsteroidal anti-inflammatory drugs (NSAIDs) and corticosteroids. Most JIA patients and their parents are educated regarding the treatment-related risk of infection (11). Therefore, an epidemic with the new coronavirus is of major concern. Families affected by JIA have been particularly impacted by this situation because of conflicting data regarding viral transmission in school by children that were initially considered superspreaders (12–16). At the onset of the sanitary crisis, the vulnerability of patients treated with disease-modifying anti-rheumatic drugs (DMARDs), nonsteroidal anti-inflammatory drugs (NSAIDS) or corticosteroids had been suggested on the basis of experience gained in other situations of viral infections and data from the first cohort studies of SARS-CoV-2-infected patients (17, 18). Despite rapid clarification from health authorities and learned French rheumatologic and pediatric societies regarding the indication to continue ongoing immunosuppressive therapy in most clinical situations, some of these scientific data and their relay through the media may have impacted the ongoing immunosuppressive treatment of JIA patients (19). In May, the French pediatric society also made recommendations about the urgent need for a return to school, highlighting the desocialization and isolation of some children and the risk of domestic violence in some cases (20).

Accordingly, our objectives were (1) to evaluate the impact of the COVID-19 pandemic on the therapeutic management of JIA, (2) the frequency of returning to school after the first lockdown period, (3) and finally the prevalence of SARS-CoV-2 infection at the time of the survey.

## Patients and Methods

We conducted a cross-sectional observational multicenter study among JIA patients. The study was proposed to French centers known for their expertise in the management of JIA patients. Eight centers participated in the study. Four were located in areas of intense viral circulation at the time of the study (Strasbourg, Nancy, Belfort and Paris), and four were located in areas of low viral circulation (Brest, Rennes, Bordeaux, and Poitiers). To be eligible for the study, JIA patients had to be under 18, a diagnosis confirmed by a clinician expert, classified by ILAR and being treated with DMARDs at the last follow-up visit. All patients and parents gave their oral consent to participate in the study, giving the exceptional situation a written consent was deemed unnecessary. This study received approval from the Brest University Hospital Ethics Committee (B2020CE.27) and was registered at ClinicalTrial.gov (NCT04407923).

A specific questionnaire was administered to patients satisfying eligibility criteria and their parents between 05/26/2020 and 08/27/2020 during face-to-face or remote consultations (Supplementary Table 1). The questionnaire was completed by either the practitioner or a study nurse. The objectives of the questionnaire were clearly explained to the patients and families to obtain accurate information. Data regarding the general characteristics of the participants, medical history, SARS-CoV-2 infection, characteristics of JIA subtypes and treatment modifications were collected.

For general characteristics and medical history, the following data were collected: sex, height, weight, urban or rural residency, smoking habits and comorbidities including diabetes, history of severe infection, and asthma. Regarding JIA characteristics, the date of diagnosis and the presence or absence of the following parameters were collected: antinuclear antibodies, rheumatoid factors, anti-citullinated protein antibodies (ACPA), erosive disease, history of prolonged corticosteroid consumption (≥5 mg per day for more than 3 months), history of uveitis, macrophage activation syndrome, patient global assessment of disease activity (0–10), and ongoing symptomatic (NSAIDs and/or corticosteroids) and DMARD treatments (conventional synthetic and biologic) at the last visit.

Collected data concerning SARS-CoV-2 infections included date of symptom onset and characterization of suspicious or RT-PCR (Reverse Transcriptase–Polymerase Chain Reaction) confirmed cases. Collected data regarding symptomatic and/or DMARD treatment modifications included date and characterization of the modifications made for each ongoing treatment, reason for treatment modification (sanitary context, infection, other) and characterization of who made the decision to modify the treatment (patient, parents, general practitioner (GP), rheumatologist, nurse, pharmacist, or other).

Regarding the return to school, the patients and/or parents were questioned about the date of the return to school and in the absence of a return to school, they were asked about the reason, if it was related to the pandemic situation, and who decided to refuse the return to school (child, parents, GP, local or administrative impossibility, or something else).

The primary objective was to determine the proportion of participants for which DMARD treatment was reduced or stopped in relation to the context of the pandemic in the absence of active infection or identify some other reason to modify the usual treatment regimen. The proportion of participants for which treatment was transiently reduced or stopped within the same period for other reasons (active infection, acute medical illness, planned surgery, and disease activity) was also collected. Secondary objectives were to determine the proportion of patients for which symptomatic treatment (corticosteroids and/or NSAIDs) was modified in relation to the sanitary crisis and to provide an overall characterization of the decision to alter DMARD and/or symptomatic treatments. The problem of returning to school was also assessed by calculating the percentage of patients who returned to school and the reason for returning or not returning. We also reported confirmed or suspicious cases of SARS-CoV-2 infections among the studied population. Data are reported as the mean ± standard deviation for continuous variables and numbers (percentages) for categorical variables.

## Results

### Characteristics of Participants

A total of 173 JIA patients were included between May and August 2020 with a mean age of 11.6 ± 4.1 years. In this sample, 69.9% (121/173) were girls. Rheumatoid factor-negative polyarticular JIA was the most common subtype of the disease, affecting 31.2% (54/173) of the participants, followed by nonextended oligoarticular JIA (24.3%). Regarding the treatments, 50% (86/172) of the patients were treated with methotrexate, and 72.5% (124/171) were treated with bDMARDS (alone or in combination with methotrexate). A total of 110/173 patients (63.6%) lived in an area of intense viral circulation. Additional characteristics of the participants are described in Table 1.

**Table 1:** Patients characteristics at inclusion.

**Patients' characteristics**		**Total**	**Hot zones**	**Others zones**
Number of patients		*N* = 173	110/173 (63.6%)	63/173 (36.4%)
Age (mean ± SD), years		11.6 ± 4.1	11.7 ± 4.1	11.6 ± 4.1
Sex (*N* = male/female)		52/121 (30.1/69.9%)	32/78 (29.1/70.9%)	20/43 (31.7/68.2%)
Disease duration (mean ± SD), months		48 (± 36)		
JIA subtypes n (%)	Systemic	15/173 (8.7%)	11/110 (10%)	4/63 (6.3%)
	Polyarticular Rheumatoid factor +	15/173 (8.7%)	8/110 (7.2%)	7/63 (11%)
	Polyarticular Rheumatoid factor -	54/173 (31.2%)	37/110 (33.6%)	17/63 (27%)
	Extended oligoarticular	18/173 (10.4%)	12/110 (10.9%)	6/63 (9.5%)
	Nonextended oligoarticular	42/173 (24.3%)	24/110 (21.8%)	18/63 (28.5%)
	Undifferentiated	2/173 (1.15%)	0/110 (0%)	2/63 (3.2%)
	Psoriasic	4/173 (2.31%)	4/110 (3.6%)	0/63 (0%)
	Enthesitis related	22/173 (12.7%)	13/110 (11.8%)	9/63 (14.3%)
Treatments n (%)	NSAIDs	24/169 (14.2%)	11/106 (10.3%)	13/63 (20.6%)
	Methotrexate	86/172 (50%)	52/109 (47.7%)	34/63 (54%)
	Biologics	124/171 (72.5%)	77/108 (71.3%)	47/63 (74.6%)
	Corticosteroids	13/169 (7.69%)	5/107 (4.7%)	8/62 (12.9%)
Comorbidities n (%)	Diabetes	2/171 (1.16%)	0/108 (0%)	2/63 (3.2%)
	Obesity	6/163 (3.68%)	3/103 (2.9%)	3/60 (5%)
	Asthma	8/171 (4.67%)	4/108 (3.7%)	4/63 (6.3%)
	Smoking	2/170 (1.17%)	1/107 (0.9%)	1/63 (1.6%)
School level (%)	Nursery	19/156 (12.17%)	12/99 (12.1%)	7/57 (12.3%)
	Primary school	43/156 (27.56%)	28/99 (28.3%)	15/57 (26.3%)
	Middle school	61/156 (39.10%)	40/99 (40.4%)	21/57 (36.8%)
	High school	33/156 (21.15%)	19/99 (19.2%)	14/57 (24.5%)
Place of residence (%)	City	78/171 (45.6%)	53/109 (48.6%)	25/62 (40.3%)
	Countryside	93/171 (54.4%)	56/109 (51.3%)	37/62 (59.6%)

*JIA, juvenile idiopathic arthritis; NSAID, nonsteroidal anti-inflammatory drug; DMARD, disease-modifying antirheumatic drug*.

### Treatment Modifications

Regarding DMARD (sDMARDS and bDMARDS) treatment, 4% (7/173) of the patients declared to have decreased or stopped their treatment in relation to the sanitary crisis (Table 2). Within the same period, 2.3% (4/173) reported DMARD treatment modifications based on medical decisions independent of the sanitary crisis (3 due to decreased disease activity and one due to intercurrent infection).

**Table 2:** Treatment modification in the JIA population.

**Treatments**		**Proportion of participants usually treated with this drug class**	**Proportion of users with corresponding treatment modification (stop or decrease) linked to the sanitary crisis**		
			**Total**	**Hot zone**	**Other zones**
DMARD treatments	Methotrexate	86/172 (50%)	2/86 (2.3%)	1/109 (0.9%)	1/63
	Other synthetic DMARD	2/171 (1.17%)	0/2 (0%)	0	0
	Biological DMARD	124/171 (72.5%)	5/124 (4.0%)	4/108 (3.7%)	1/63 (1.5%)
Symptomatic treatments	Corticosteroids	13/169 (7.69%)	0/13 (0%)	0	0
	NSAIDs	24/169 (14.2%)	3/24 (12.5%)	0	3/63 (4.7%)

*DMARD, disease-modifying antirheumatic drug*.

Regarding NSAIDs and corticosteroids, 8.1% (3/37) of those patients taking NSAIDs or corticosteroids declared to have reduced or stopped their treatment in relation to the sanitary crisis (only patients taking NSAIDs), and one more patient stopped corticosteroid treatment for medical reasons.

Concerning disease activity, we did not find a significant difference in disease activity between children who modified DMARD treatment in relation with the sanitary health crisis (mean visual analog scale (VAS): 25.8/100) compared to children who did not modify DMARD treatment (mean VAS: 11.6/100, *p* = 0.47).

### Characterization of the Decision to Modify Treatment

Among the seven patients for whom DMARD treatment was modified in relation to the sanitary crisis, the decision was made by the patient or the parents in 85.7% of cases (6/7) and made by the specialist for one patient. Among the three patients who modified their NSAID treatment in relation to the sanitary crisis, decisions were made by the patient or the parents in 2 cases.

### Return to School

A total of 57.6% (98/170) of families described a fear of returning to school, and 49.1% (81/165) of the patients did not return to school. It was the consequence of the child's decision in 19.75% (16/81) of cases, a parental decision in 48.1% (39/81) of cases, and due to a local or administrative impossibility in 22.2% (18/81) of cases. More detailed data are described in Table 3.

**Table 3:** Returning to school in the JIA population.

	**Total**	**Hot zone**	**Other zones**
Reluctance to return to school	98/170 (57.6%)	64/109 (58.7%)	34/61 (55.7%)
No resumption of schooling	81/165 (49.1%)	50/107 (46.7%)	31/58 (48.4%)
Patient decision	16/81 (19.75%)	11/50 (22%)	5/31 (16.1%)
Parent decision	39/81 (48.1%)	24/50 (48%)	15/31 (48.4%)
Specialist decision	3/81 (3.7%)	1/50 (2%)	2/31 (6.4%)
Administrative impossibility	18/81 (22.2%)	12/50 (24%)	6/31 (19.3%)
General practitioner	1/81 (1.2%)	1/50 (2%)	0
Unknown	4/81 (4.9%)	1/50 (2%)	3/31 (9.6%)

### Comparison Between Areas of Intense Viral Circulation and Other Areas

We compared the frequency of treatment modification and frequency of return to school in the two areas.

A total of 71.4% (5/7) of the patients reporting a change in their DMARD treatment due to the sanitary health crisis were patients from an area of intense viral circulation. One hundred percent (3/3) of patients reporting a change in their NSAID treatment due to the sanitary health crisis were patients from the other zones (Table 2). There is no significant difference in DMARD treatment changes related to the sanitary health crisis between these two groups, with 5/110 in the hot zone group and 2/63 in the other group (*p* = 1).

We did not find any significant difference in the reluctance to return to school (58.7% in areas of intense viral circulation and 55.7% in the other areas) or in the resumption of schooling; 46.7% of children did not return to school in areas at greater risk of contamination, while 48.8% did return to school in the other areas (Table 3).

### Frequency of COVID-19

A total of 4.6% (8/173) of JIA patients reported a history of possible SARS-CoV-2 infection at the time of the survey. Two patients were diagnosed as positive for SARS-CoV-2: one case was confirmed with RT-PCR, and one other case was confirmed by serological testing. The other patients were not tested or had negative results.

## Discussion

The objectives of our study were to evaluate the consequences of fear on therapeutic compliance in an epidemic context and a new concept of return to school after lockdown. This large survey was based on questionnaires but provides a global picture of patients' and parents' attitudes. Our results highlight the fact that, in the end, a majority of patients declared to have unchanged their treatments on the one hand and to be afraid to return to school on the other hand. These data were not correlated to hot spot zones but were a global attitude in France.

In our study, we unexpectedly showed that patients did not modify their immunosuppressive treatment in a large majority of cases. For those who changed their treatment, independent of a medical decision, the reason was clearly fear of the patients due to the global context. In our cohort, only 14 patients reduced or stopped their treatment at the time of our study, and among them, only nine patients declared that their modifications of treatment were related to the health sanitary crisis (six patients with DMARD, two patients with NSAIDs and one patient for both) and not to a medical recommendation. Therefore, our results reflect good adherence of the patient/parents to their immunosuppressive treatments. This seems to be consistent with other studies, showing good adherence to biological treatments (21–24) and in this unprecedented context, in comparison to patients with other chronic non-respiratory pathologies treated with immunosuppressors, JIA patients seems to be compliant with their treatment. In fact, children with inflammatory bowel disease did not discontinue their immunomodulatory treatments (25), patients with rheumatoid arthritis declared a modification in their treatment in 11% of cases (26), and patients with systemic lupus modified their treatment in 10% of cases (27). Of course, these data must take into account the self-report aspect of our study and the difficulty of measuring therapy adherence. In chronic pediatric diseases, therapeutic adherence depends not only on the patient's behavior but also on their parents, whose role is essential. Adherence (also named compliance or observance) to treatment is a complex notion that is difficult to measure (21–23). Direct assessment criteria, such as biological drug assays, are invasive and not easy to perform in routine practice. Therefore, indirect measurements have been developed, frequently based on self-administered questionnaires. For example, the Child Adherence Report Questionnaire (CARQ) (22), the Parent Adherence Report Questionnaire (PARQ) (28), or the Morisky questionnaire have been used to evaluate children's therapy adherence (29). However, this study describes the therapeutic modifications that can be highlighted during a classic interview with patients but does not take into account the more complex problem of noncompliance.

The results of the study are quite similar in all of France. This suggests that the local viral circulation does not influence patients' attitudes. The fear of returning to school was not greater in areas of intense viral replication.

Moreover, our study suggests, as others have, that at the time we questioned patients (during the first wave and in months that followed), there was a low rate of contamination during the first wave, with only two patients diagnosed positive for SARS-CoV-2, which is corroborated by several studies showing no or few cases of COVID-19 (30–32). The small number of patients diagnosed positive for SARS-CoV-2 could be explained by the low number of tests performed on children at beginning of the pandemic, but also because population avoided going to hospital because of fear. As expected, the frequency of comorbidities was rare, and few children benefited from long-term treatment with corticosteroids or NSAIDs.

One of the most interesting results was about returning to school. In France, school reopening plans were not based on large testing programs or exhaustive screening for COVID-19 symptoms but on physical distancing measures, masks, hygiene and cohorting students in smaller groups. Although it may have been important for the parents that their children return to school, 57.6% of the patients were anxious, and 49% of the JIA patients did not return to school due to anxiety of the parents regarding COVID-19. In comparison with national French data from 24 June 2020, 80% of schoolchildren (primary and secondary), have returned to school (33). Not return to school was related to parents' decisions in 39/81 (48.1%) of cases but also to administrative decisions in 18/81 (22.2%) of cases and sometimes based on difficulties for parents in organizing themselves regarding their own jobs. This clearly revealed that the balance between risk and benefit for return to school was negative for the parents and this is corroborated by a recent study, showing better adherence to isolation measures in patients with rheumatic diseases (34).

Our study has some limitations: questionnaires were given to the patients at the end or during the outpatient visit or during a phone call, and this questionnaire was only self-reported, which could have influenced the results. In some cases, patients could have hesitated to mention their own attitude regarding their treatment and may partly explain why we have such a low rate of reported treatment modification. Moreover, this questionnaire is not a validated questionnaire for measuring treatment compliance. All subtypes of JIA were not equally represented: we had a low number of patients with severe disease, such as systemic JIA, where most patients would have severe comorbidities related to COVID-19, such as high doses of corticosteroids. As we included only patients with immunosuppressive treatment, we cannot extrapolate to JIA treated only with NSAIDs. Moreover, oligoarticular JIA is usually the most frequent type of JIA but not in our study, which was probably due to the need to be receiving DMARD treatment to be included, while various types of oligoarticular JIA are treated with NSAIDs only. Perhaps a larger sample that included these patients could have shown different results regarding adherence to treatment. In our population, few patients tested positive for SARS-CoV-2, probably due to a low number of tests carried out on the children in the first wave. On 9 September 2020, the French Society of Pediatrics proposed some new recommendations, particularly more regular screening of patients over 6 years of age (35).

In our study, we clearly demonstrated that a large majority of JIA patients did not modify their immunosuppressive treatment despite fears involving the pandemic context, but the confidence in returning to school was low considering the risk of COVID-19. JIA patients were rarely affected by COVID-19 during the first wave. Therefore, future efforts must focus on clear programs for returning to school.

## Data Availability Statement

The raw data supporting the conclusions of this article will be made available by the authors, without undue reservation.

## Ethics Statement

The studies involving human participants were reviewed and approved by Brest University Hospital Ethics Committee (B2020CE.27). Written informed consent from the participants' legal guardian/next of kin was not required to participate in this study in accordance with the national legislation and the institutional requirements.

## Author Contributions

BQ: investigation, formal analysis, writing original draft, review, and editing. IL, AL, PP, JM, OR, CS, VD, LR-S, CB, IK-P, and EG: investigation, writing review, and editing. DG: conceptualization, methodology, formal analysis, writing review and editing, visualization, and supervision. VD-P: conceptualization, methodology, writing review and editing, visualization, supervision, and project administration. All authors contributed to the article and approved the submitted version.

## Conflict of Interest

The authors declare that the research was conducted in the absence of any commercial or financial relationships that could be construed as a potential conflict of interest.

## Publisher's Note

All claims expressed in this article are solely those of the authors and do not necessarily represent those of their affiliated organizations, or those of the publisher, the editors and the reviewers. Any product that may be evaluated in this article, or claim that may be made by its manufacturer, is not guaranteed or endorsed by the publisher.
