# Supplementary material for: Juvenile Idiopathic Arthritis and COVID-19 Pandemic: Good Compliance With Treatment, Reluctance to Return to School
Source: Front Med (Lausanne). 2021 Nov 12;8:743815. doi: 10.3389/fmed.2021.743815 (PMC8632709; doi:10.3389/fmed.2021.743815)
Supplement: Supplementary file 1 [file Data_Sheet_1.PDF]

Supplementary data:

Questionnaire for the impact of COVID-19 in JIA patients

Original language (French)	English translation
1. **Vérification des critères d'éligibilité :**<br>○ Age inférieur ou égal à 18 ans<br>○ AJI confirmée (critères d'Edmonton)<br>○ AJI bénéficiant d'un traitement de fond<br>○ Patient représentant légal acceptant de participer à la recherche	1. **Verification of eligibility criteria:**<br>○ Age up to 18 years<br>○ Confirmed JIA (Edmonton criteria)<br>○ JIA with background treatment<br>○ Patient as legal representative agreeing to participate in the research
2. **Identification du patient :**<br>○ Moi et année de naissance :<br>○ Date de la consultation :<br>○ Département du lieu de résidence :	2. **Patient identification:**<br>○ Month and year of birth:<br>○ Date of consultation:<br>○ Department of residence
3. **Type d'AJI**<br>○ AJI systémique : Oui / Non<br>○ AJI oligo-articulaire extensive : Oui / Non<br>○ AJI oligo-articulairenon extensive : Oui / Non<br>○ AJI polyarticulaire séronégative : Oui / Non<br>○ AJI polyarticulaire séropositive : Oui / Non<br>○ AJI liée aux enthésites : Oui / Non<br>○ AJI psoriasique : Oui / Non<br>○ AJI indifférenciée : Oui / Non	3. **Type of JIA**<br>○ Systemic JIA: Yes / No<br>○ Extended oligoarticular JIA: Yes / No<br>○ Non extended oligoarticular JIA: Yes / No<br>○ Polyarticular Rheumatoid factor +: Yes / No<br>○ Polyarticular Rheumatoid factor -: Yes / No<br>○ Enthesitis-related JIA: Yes / No<br>○ Psoriatic JIA: Yes / No<br>○ Undifferentiated JIA: Yes / No
4. **Caractéristiques générales et antécédents**<br>○ Sexe : Fille / Garçon<br>○ Taille :<br>○ Poids :<br>○ Lieu de résidence : Rural / Urbain<br>○ Tabagisme actif : Oui / Non<br>○ Asthme : Oui / Non<br>○ Diabète : Oui / Non<br>○ Antécédent d'infection sévère : Oui / Non	4. **General characteristics and history**<br>○ Sex: Female / Male<br>○ Height:<br>○ Weight:<br>○ Place of residence: Rural / Urban<br>○ Active smoking: Yes / No<br>○ Asthma: Yes / No<br>○ Diabetes: Yes / No<br>○ History of severe infection: Yes / No
5. **Notion d'infection Covid-19**<br>○ Notion d'infection Covid-19 : Oui / Non<br>○ Si oui : Cas suspect / Cas confirmé<br>○ Date du début des symptomes :	5. **Notion of Covid-19 infection**<br>○ Notion of Covid-19 infection: Yes / No<br>○ If yes: Suspected case / Confirmed case<br>○ Date of onset of symptoms:
6. **Caractéristiques de l'AJI**<br>○ Année du diagnostic :<br>○ Date de la dernière consultation :<br>○ Factuers antinucléaires : Oui / Non<br>○ Facteur rhumatoïde : Oui / Non<br>○ ACPA: Oui / Non<br>○ Antécédent d'uvéite: Oui / Non<br>○ Antécédent de SAM: Oui / Non<br>○ Maladie érosive : Oui / Non<br>○ Antécédent de corticothérapie prolongée* : Oui / Non *(>= à 5mg par jour pendant plus de 3 mois)	6. **Characteristics of JIA**<br>○ Year of diagnosis:<br>○ Date of last consultation<br>○ Antinuclear factors: Yes / No<br>○ Rheumatoid factor: Yes / No<br>○ ACPA: Yes / No<br>○ History of uveitis: Yes / No<br>○ History of MAS: Yes / No<br>○ Erosive disease: Yes / No<br>○ History of prolonged corticosteroid therapy*: Yes / No *(>= 5mg per day for more than 3 months)
7. **Traitements habituels de l'AJI**<br><br>Traitements symptomatiques :<br>○ Corticotïdes : Oui / Non<br>○ AINS : Oui / Non<br><br>Traitements de fond :<br>○ Méthotrexate : Oui / Non<br>○ Hydroxychloroquine : Oui / Non<br>○ Autre sDMARD : Oui / Non<br>○ bDMARD : Oui / Non<br>○ tsDMARD : Oui / Non	7. **Usual treatments for JIA**<br><br>Symptomatic treatments:<br>○ Corticosteroids: Yes / No<br>○ NSAIDs: Yes / No<br><br>Background treatments:<br>○ Methotrexate: Yes / No<br>○ Hydroxychloroquine: Yes / No<br>○ Other sDMARD: Yes / No<br>○ bDMARD: Yes / No<br>○ tsDMARD: Yes / No

8. **Allègement du traitement habituel du fait de la crise sanitaire** :<br><br>Allègement du traitement sur la période : Oui / Non<br><br>Si oui :<br>o Allègement en lien avec la crise sanitaire : Oui / Non<br>o Allègement pour un évènement infectieux intercurrent (autre que COVID)<br>o Autre :<br><br>Prise de décision de l'allègement par :<br>o Patient/parents<br>o Médecin généraliste<br>o Pédiatre<br>o Rhumatopédiatre<br>o Pharmacien<br>o Autre :<br><br>Nature de l'allègement à préciser :<br>o AINS : Oui / Non<br>o Corticoïdes : Oui / Non<br>o Méthotrexate : Oui / Non<br>o Hydroxychloroquine : Oui / Non<br>o Autre sDMARD : Oui / Non<br>o bDMARD : Oui / Non<br>o tsDMARD : Oui / Non<br><br>Si oui :<br>o AINS : Diminution / Suspension<br>o Corticoïdes : Diminution / Suspension<br>o Méthotrexate : Diminution / Suspension<br>o Hydroxychloroquine : Diminution / Suspension<br>o Autre sDMARD : Diminution / Suspension<br>o bDMARD : Diminution / Suspension<br>o tsDMARD : Diminution / Suspension<br><br>Augmentation compensatrice des co-traitements antalgiques :<br>o Antalgiques de pallier 1 : Oui / Non<br>o Antalgiques de pallier 2 : Oui / Non<br>o Antalgiques de pallier 3 : Oui / Non<br><br>9. **Retour à l'école selon les recommandations ministérielles :**<br>o Réticence à la reprise scolaire du fait du contexte épidémique : Oui / Non<br>o Date prévue de reprise :<br>o Niveau scolaire :<br>o Le patient a t'il repris : Oui / Non<br><br>Si non, préciser le ou les motifs :<br>o Décision personnelle (patient)<br>o Décision des parents<br>o Décision du médecin traitent<br>o Décision du rhumatopédiatre referent<br>o Impossibilité locale ou administrative<br>o Poussée de AJI<br>o Autre raison de santé:	8. **Reduction of usual treatment due to the health crisis**:<br><br>Reduction in treatment over the period: Yes / No<br><br>If yes:<br>o Modification related to the health crisis: Yes / No<br>o Modification for an intercurrent infectious event (other than COVID)<br>o Other reason:<br><br>Decision to relieve by:<br>o Patient/parents<br>o General practitioner<br>o Paediatrician<br>o Rheumatologist<br>o Pharmacist<br>o Other:<br><br>Nature of reduction to be specified:<br>o NSAIDs: Yes / No<br>o Corticosteroids: Yes / No<br>o Methotrexate: Yes / No<br>o Hydroxychloroquine: Yes / No<br>o Other sDMARD: Yes / No<br>o bDMARD: Yes / No<br>o tsDMARD: Yes / No<br><br>If yes:<br>o NSAIDs: Decrease / Suspension<br>o Corticosteroids: Decrease / Suspension<br>o Methotrexate: Decrease / Suspension<br>o Hydroxychloroquine: Decrease / Suspension<br>o Other sDMARD: Decrease / Suspension<br>o bDMARD: Decrease / Suspension<br>o tsDMARD: Decrease / Suspension<br><br>Offsetting increase in analgesic treatments:<br>o Grade 1 analgesics: Yes / No<br>o Grade 2 analgesics: Yes / No<br>o Grade 3 analgesics: Yes / No<br><br>9. **Return to school according to ministerial recommendations:**<br>o Reluctance to return to school due to the epidemic: Yes / No<br>o Expected date of return to school:<br>o School level:<br>o Has the patient returned to school: Yes / No<br><br>If not, specify the reason(s):<br>o Personal decision (patient)<br>o Parents' decision<br>o Decision of the treating physician<br>o Decision of the referring rheumatologist<br>o Local or administrative impossibility<br>o Outbreak of JIA<br>o Other health reason:

Table 1: Specific questionnaire administered to JIA patients during face-to-face or remote consultations.
